# Editorial

**Published:** 1890-03

**Authors:** 


### Editorial.

### A WORD TO EXAMINING BOARDS.

As the time is drawing near when the graduates of the various colleges will go forth, with newly acquired diplomas, and will have to appear in many states before an Examining Board, in order to obtain the right enter upon the practice of their profession, we cannot refrain from a few words on the subject. We would, in the beginning, preface these remarks with the statement that we are in favor of Examining Boards. There is no doubt in the minds of all the intelligent medical men that their influence has been for the good of the profession, while at the same time there is also little doubt that, in many cases, they wrought injustice to both colleges and graduates.

We have only two complaints to make against many of the examination papers that have come before us, and they are: first, that some of them are unreasonably rigid; and second, there are many instances in which the questions propounded are of not such a nature as to test the actual capability of the candidate.

The Board of Examiners is, as a rule, composed of men who have no practical experience in teaching or examining, and men who are of more than ordinary ability. Naturally, they fix their standard at a high point, and they lose sight of the fact that, after all, the graduate is only beginning his career. Manifestly it is not reasonable to demand of him a standard to which not one in twenty of the practitioners in his community attain. We remember as an instance of this, that a few years since the Medical Examining Board of Virginia put before the applicants for license an array of questions that would have rejected almost any man who had been in the actual practice of his profession for any length of time, and it was very properly protested against by the medical press of this country. It was simply unreasonable to base a man's right to prosecute his work in the state on his ability to stand an examination on chemistry that was only fit for a professional chemist. And so in many cases, there has been the tendency to subject the candidate to a rigid line of questions, that in the mind of one accustomed to examining, would not be a fair test of his general practical knowledge. After all, there are many things that go to make up the successful practitioner of medicine, and some allowance should be made for the more practical bent of a man's disposition. Again, the examiner should not expect that the new graduate should know as much as himself.

In the matter of questions that are not representative of the science of medicine, and above all of its practical side, we would refer to an examination that was given a few months since in the State of Alabama, notorious throughout the country for the stand that it has taken in raising the standard of its practitioners. A man who had been a hard and thorough student, and who had taken a post graduate course for a session after his graduation, appeared before a Board for license in the State. He was sufficiently well posted to be a matter of pride to his alma mater, and he prepared himself for a rigid examination, especially in anatomy. Imagine his consternation when he was confronted with such questions as, "give the origin and insertion of the tensor palati muscle," "describe the longest part of the long issimis dorsi," and many of the same nature, instead of selecting some of the many hundreds of practical subjects with which he has to deal in the work of every-day life. In other departments were found such questions as, "how long can a child live after birth without breathing," etc.

No one can say that a school that has made an earnest effort to give especial prominence to the more valuable and practical parts of medicine, has not the right to protest against their diplomas being subjected to the humiliation of not being accepted in the State, when it is done on such catch questions as these that are more applicable to the boy in his school physiology than a man who is entering upon his career in the practice of medicine.

No honest college should object to the appearance of a graduate before a Board, but they have the right to demand that he shall be given plain justice. That there should be sixty per cent. of the graduates of the reputable colleges of this country unfit to practice medicine in the State of Virginia, seems incredible, yet that is the proportion at their last examination that was found so. To most of the schools that are honest in the effort to do good work, this is a sad blow, and shows that somebody has undoubtedly been badly out of the way.

### HOW TO BECOME AN ARMY SURGEON.

The circular recently issued by the Surgeon General, calling upon the aspirants for medical service in the army, is accompanied by a copy of the questions that were pronounced at the last sitting of the Board, and as some of the lines followed, are of such an encouraging nature to the man who has been for a number of years devoting himself to the study of legitimate medicine, we take pleasure in reproducing them, and reverently trust that they may encourage and cheer the lonely heart of some poor soul longing for shoulder-straps.

If you can tell the "principal victories won by Peter the Great," or to go back further, make running comments on all the great and small gods that were to be found in ancient mythology, you may be all right. Yet you are required to know "the most direct route from St. Louis to the City of Mexico," as well as give an account of Mohammed and his doings. These, and such questions as "name principal works of George Elliot," etc., go to make up what may be termed the "trimmings," after which comes the regular tilt on topics medical. Altogether it behooves him who would ascend the outer walls to gird up his loins and furnish up his general knowledge, or otherwise he will be among the twenty-eight out of thirty who will go in the same steps as those of last year.

### PHYSICAL EDUCATION.

We have noticed with pleasure the increased interest in physical education for women. This is particularly needed in the South. Climate and general surroundings all tend to a general relaxation, and with it faulty development.

It must always be remembered that sound minds and sound bodies go together.

Every school should have its teacher of Physical Education.

Not simply a teacher of calaisthenics, but one who understands the needs and requirements of the different students. Exercise imperfectly used is worse than none, and often causes incomparable injury.

---

B. T. Barnum has said that people like to be humbugged, and surely it does seem so. Look at our daily papers and the advertisements. Drs. Tom, Dick and Harry, all give written guaranties to cure all diseases that the flesh is heir to, and actually use the papers as reference. Again all sorts and kinds of patent nostrums are given the support of our secular press.

It does seem strange that a man will carefully examine all machinery and all sources of power to be used in his business; but when it comes to himself he accepts with closed eyes, and I might say ears all in shape of medicine for his own use.

Are not the physicians to a certain degree responsible for this, many being ready and willing to give their signature to anything that presents. This arises from two facts, either that overwhelming desire to see their name in print, or else their ignorance, not being able to write prescriptions, so are willing to take whatever is given them as a God sent help in time of need.

---

### RINGWORM.

Ringworm of the body is generally very amenable to treatment, judging from the numerous domestic remedies which act so successfully. Sometimes, however, an obstinate case is encountered, and recourse is had to the physician. In such cases a rapid cure is desirable, and the application of the following application, once daily, for two or three consecutive days, will generally prove successful:

℞ Hydrarg. Bichloridi - 1 grain.
Tinct. Benzoin. Comp. - 1 ounce.
M. Sig.: Paint over affected part.

Care should be exercised not to paint too large a surface, as the above mixture is toxic. If an excoriation should exist, it should not be applied, as it is irritating to the wounded integument.—*Medical Chips.*

